# Inflammatory Bowel Disease: The Emergence of New Trends in Lifestyle and Nanomedicine as the Modern Tool for Pharmacotherapy

**DOI:** 10.3390/nano10122460

**Published:** 2020-12-09

**Authors:** Eden Mariam Jacob, Ankita Borah, Sindhu C Pillai, D. Sakthi Kumar

**Affiliations:** Bio-Nano Electronics Research Centre, Graduate School of Interdisciplinary New Science, Toyo University, Kawagoe, Saitama 350-8585, Japan; s4r101800020@toyo.jp (E.M.J.); ankita@toyo.jp (A.B.); s4r101700018@toyo.jp (S.C.P.)

**Keywords:** inflammatory bowel disease (IBD), Crohn’s disease (CD), ulcerative colitis (UC), gut microbiome, immunity, nutrition, probiotics, nano-drug delivery

## Abstract

The human intestine, which harbors trillions of symbiotic microorganisms, may enter into dysbiosis when exposed to a genetic defect or environmental stress. The naissance of chronic inflammation due to the battle of the immune system with the trespassing gut bacteria leads to the rise of inflammatory bowel disease (IBD). Though the genes behind the scenes and their link to the disease are still unclear, the onset of IBD occurs in young adults and has expanded from the Western world into the newly industrialized countries. Conventional drug deliveries depend on a daily heavy dosage of immune suppressants or anti-inflammatory drugs targeted for the treatment of two types of IBD, ulcerative colitis (UC) and Crohn’s disease (CD), which are often associated with systemic side effects and adverse toxicities. Advances in oral delivery through nanotechnology seek remedies to overcome the drawbacks of these conventional drug delivery systems through improved drug encapsulation and targeted delivery. In this review, we discuss the association of genetic factors, the immune system, the gut microbiome, and environmental factors like diet in the pathogenesis of IBD. We also review the various physiological concerns required for oral delivery to the gastrointestinal tract (GIT) and new strategies in nanotechnology-derived, colon-targeting drug delivery systems.

## 1. Introduction

**Figure 1:**
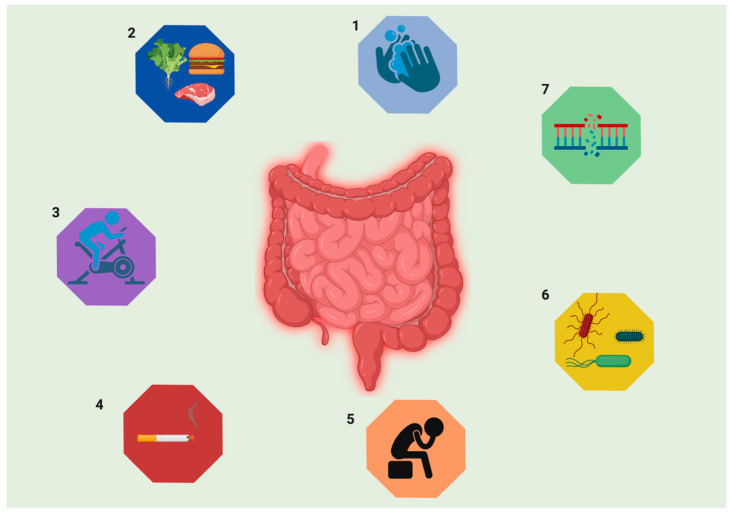
The chronic inflammation of the intestines in inflammatory bowel disease (IBD) can be caused by various factors including environmental triggers that can be classified into five major causes: (1) poor basic hygiene, (2) unbalanced dietary intake, (3) lack of physical exercise, (4) increased smoking (in the case of Crohn’s disease), and (5) stress. The internal factors include (6) gut microbiota dysbiosis in the intestine and (7) unpredictable genetic modification. Image developed using Biorender.com.

Inflammatory bowel disease (IBD) represents an umbrella term for the chronic remission and relapse of immunologically-mediated idiopathic diseases. IBD is generally diagnosed under two major classifications as Crohn’s disease (CD) and ulcerative colitis (UC) with significantly contrasting etiologies [1]. Multiple studies over the decades have still remarkably left the pathogenesis of the diseases an unresolved mystery. CD tends to occur in any part of the gastrointestinal tract (GIT) and is associated with complications [2], whereas UC, on the other hand, is strictly restricted to the inflammation of the colon [3]. The onset of the diseases is marked at young adulthood [4] in genetically susceptible individuals responding to commensal microbes or environmental cues [5] like poor hygiene [6,7], unbalanced dietary intake [8], a lack of physical exercise [9], smoking, and stress [10] (Figure 1). The downhill trajectory on the quality of life demands a heavier burden of therapy and hospitalization due to the delayed diagnosis of the disease. An improved understanding of the disease mechanisms from a molecular to a higher organ level can attenuate the misconceptions in its diagnosis and treatment. The identification of IBD as a polygenic disease genetics, along with the identification of commensal microflora participation in gastrointestinal environments, has led to an extravagant immune response in the gut [11]. IBD patients have an increased risk linked to colon cancer, which is the third most-common one globally [12] due to the development of chronic inflammation that is characterized by massive immune filtration and immune-mediated tissue destruction [13]. Sporadic colorectal cancers and colitis-associated cancers mostly develop due to two main drivers of carcinogenesis–immunosuppression and inflammation [14]. Chronic inflammation is believed to trigger colorectal cancer, with oxidative stress-induced DNA damage that can result in the activation of pro-carcinogenic genes and the silencing of the tumor suppressor pathway [15]. Altered microbiota that can contribute to chronic inflammation-producing carcinogenic factors have become the subject of intense research.

Recently, due to increased improvements in diagnosis and treatment, including the use of immunosuppressants, has reduced the hospitalization and surgery of patients with IBD [16]. Anti-inflammatory drugs including 5-aminosalicylic acid (5-ASA), thiopurines, and anti-tumor necrosis factor (TNF)-α used in the treatment of IBD have also reduced colitis-associated cancer and have been firmly established as chemo-preventive [17]. However, the extensive use of thiopurines in anti-inflammatory drugs has had conflicting results, sometimes leading to the development of lymphoma [18]. The inter-patient variability and inconsistent efficacy of the drug calls for advancements in targeted drug delivery systems [19]. Nanotechnology has been upraised in the pharmaceutical field for climbing barriers in the crafted delivery of hydrophobic drugs with a reduced dosage and a high efficiency, thereby minimizing the systemic toxicities that were once a burden [20]. The nanoparticle delivery system has not only uplifted conventional delivery systems but also aided in the development of new therapeutic drugs [21]. The usage of nanomedicine in oral delivery significantly enhances the biodistribution of therapeutics to the colon and also focuses on the cellular uptake of the drug within the diseased cells without harming the healthy tissues [21]. This review focuses on the pathophysiology of IBD through its genetics, microbial, and nutritional factors; the physiological consideration for oral delivery; new strategies in the nano-drug delivery systems for colon targeting through oral routes; and the future direction of this research.

## 2. Epidemiology of IBD

IBD has evolved into a global disease with great variations affecting not only the highly developed countries in North America and Europe but also the newly industrialized countries in Asia [22,23]. In Europe, the incidence of UC is 24.3–505 per 100,000 person-years, and that of CD is 12.7–322 per 100,000 person-years. Meanwhile, in North America, the prevalence of UC is 19.2–249 per 100,000 person-years and that of CD is 20.2–319 per 100,000 person-years. In Canada alone, the incidence of IBD is 18.7–28.3 per 100,000 person-years [24]. The cases of IBD in Asia and the Middle East are 6.3 per 100,000 person-years for UC and 5.0 per 100,000 person-years for CD [25]. This increase in the occurrence of IBD globally suggests the influence of environmental factors like diet, hygiene, pollutants, and microflora, along with genetic variations [22]. IBD can be diagnosed at any age from infancy to octogenarian. In Table 1, a comparative analysis between CD and UC is depicted.

## 3. Genetic Factors

Genome-wide association studies (GWAS) and candidate gene studies in UC have been able to identify 18 susceptible loci. A meta-analysis conducted by Anderson C.A. et al. [33] confirmed over 99 risk loci for IBD including 28 of the risk loci that are shared between CD and UC. An analysis of genes and genetic loci unraveled the role of several pathways in IBD that are crucial in maintaining intestinal homeostasis, including maintaining physical and chemical barrier functions, activating the defense system against pathogenic microbes, reactive oxygen species (ROS), endoplasmic reticulum (ER) stress, and metabolic pathways accompanied by cellular homeostasis [2]. The first gene to be associated with IBD was the nucleotide-binding oligomerization domain-containing protein 2 (NOD2) [34]. The NOD-like receptors (NLRs) are a class of intercellular innate immune proteins that achieve a host defense mechanism by recognizing conserved microorganism-associated molecular patterns (MAMPs). NOD2/caspase recruitment domain containing protein CARD15 associated with intestinal epithelial cells and Paneth cells responds to intracellular fragments of bacterial peptidoglycans that contain muramyl dipeptide (MDP) and initiate an innate and adaptive immune response [35]. NOD2 is also associated with the recruitment of receptor-interacting protein-2 (RIP2) that activates nuclear factor kappa B (NF-κB) pathways in epithelial cells and macrophages [36]. The bacterium and its associated viral infections can activate NF-κB signaling, resulting in the expression of various pro-inflammatory immune factors like tumor necrotic factor (TNF), interleukin 6 (IL-6), β-chemokine ligand-2 (CCL-2), neutrophil chemoattractant cysteine-X-cysteine (CXC)-chemokine ligand 8 (CXCL8/IL8), CXCL2, and antimicrobial factors like defensin [37], innate lymphoid, and myeloid cells. NOD2-deficient mice were susceptible to various bacteria like *Staphylococcus* spp., *Listeria* spp., *Citrobacterium* spp., and *Escherichia coli*. [38].

**Figure 2:**
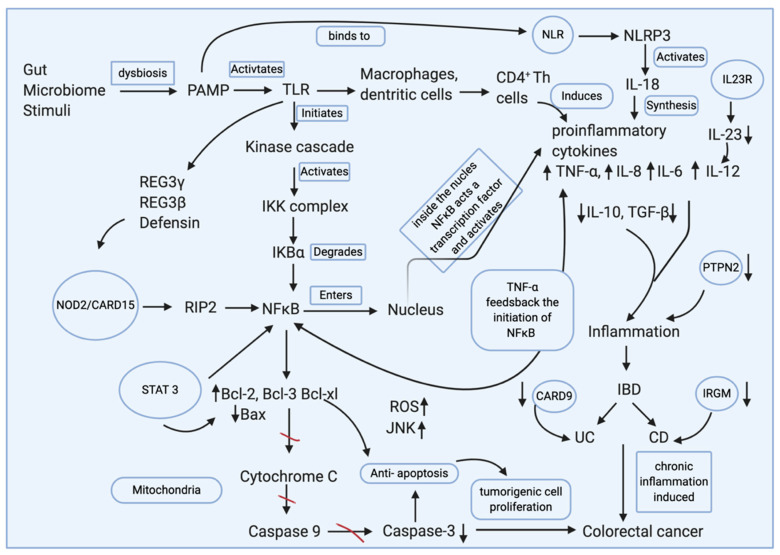
Various gut microbial stimuli due to dysbiosis can cause the initiation of inflammation in the intestine. The release of the proinflammatory cytokines inducing inflammation through various pathways assures the destruction of pathogenic microbes, and homeostasis is maintained when the resolution phase is used for controlling the inflammation and damage repair. Dysbiosis, combined with genetic defects, can omit the resolution phase with continuous chronic inflammation leading to the introduction of IBD. The pathogen-associated molecular pattern (PAMP) releases can activate the Toll-like receptors (TLRs) to initiate the regenerating islet-derived protein (REG)3γ, REG3β, and defensin that promotes the nucleotide-binding oligomerization domain-containing protein (NOD2) to release nuclear factor kappa B (NFκB). A PAMP-activated TLR can also instigate the canonical pathway for the release of NFκB through the activation of the kinase cascade that in turn activates the IKB kinase (IKK) complex where the degradation of IKKβ can release NFκB into the nuclease. The NFκB can act as a transcription factor in the production of various proinflammatory cytokines like tumor necrosis factor (TNF)-α, interleukin (IL)-8, and IL-6. TNF-α can provide feedback for the further initiation of NFκB. PAMP can also bind to the nod-like receptor (NLR) gene, the NLR family pyrin domain containing 3 (NLRP3) that activates IL-18 that can synthesize other cytokines. A defect in the IL23R gene is responsible for the depletion of IL-23 that can be involved in the upregulation of IL-12 that stimulates inflammation. Chronic inflammation can lead to colorectal cancer, which is also influenced by the activation of NFκB. The signal transducer and activator of transcription 3 (STAT3), along with NFκB, can upregulate the antiapoptotic proteins B-cell lymphoma (Bcl)-2, Bcl-3, and B-cell lymphoma-extra-large (Bcl-xl), and it can diminish the apoptotic Bcl-2 associated X protein (Bax). This can irregulate caspase activity, thereby increasing the tumorigenic proliferation of susceptible cells developing into colorectal cancer. Image developed using Biorender.com.

NF-κB enacts a crucial role in linking chronic inflammation to cancer developments. External stimuli like microbial or food antigens can cause the release of NF-κB by the degradation of the nuclear factor of kappa light polypeptide gene enhancerin B-cell inhibitor, beta (IκB) protein that regulates the NF-κB pathway. NF-κB translocates to the nucleus and regulates the expression of specific genes that are typically involved in immune responses, inflammatory responses, and cell growth control [39]. The triggering of inflammatory factors like TNFα and IL-6 can lead to extreme tissue damage due to uncontrollable inflammation. This promotes the activation of adhesion proteins, chemokines, and inhibitors of apoptosis that support cell survival—promoting the development of colitis-associated colorectal cancer [40]. NF-κB has been known to affect apoptosis by regulating anti-apoptotic proteins, prolonging c-Jun N-terminal kinase (JNK) activation, and accumulating ROS [41]. Therefore, the inactivation of NF-κB can attenuate inflammation-associated cancers [42] (Figure 2).

## 4. Gut Microbiome and Immunity

**Figure 3:**
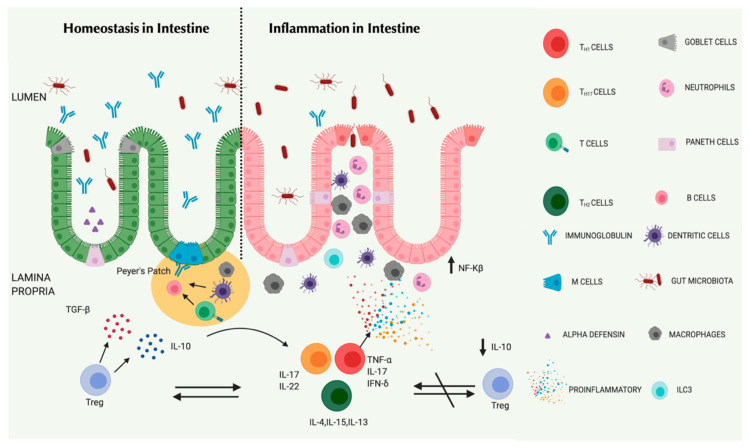
During the homeostasis of the intestine, the different layers of physical barriers along with chemical barriers are alerted to any presence of microbial invasion. The goblet cells release mucin 2 (MUC2), along with the activation of macrophages and dendritic cells present in the lymphoid tissues called Peyer’s patches covered by microfold (M) cells receiving antigens for testing, which triggers the release of proinflammatory cytokines. The Paneth cells also release α-defensins that are microbicidal. T regulatory (T_reg_) cells release IL-10 that helps in maintaining tissue homeostasis. Innate lymphoid cells (ILC) are capable of mirroring T helper (T_H_) cells, and ILC3 and T_H17_ cells produce IL-22, which is responsible for maintaining the host–microbiota balance by promoting the fucosyltransferase 2 (FUT2)-mediated fucosylation of epithelial glycans to support the symbiotic microbiota and ward off pathogenic species. In the case of IBD, the inflammation of the intestines occurs due to certain genetic defect or environmental variation that can lead to the dysbiosis of the microbial community, which, in turn, causes the T_H_ cells to produce proinflammatory cytokines that tell NF-κB to bind to TNF receptors in activating the kinase cascade that further produces more inflammatory cytokines. IL-17-stimulated neutrophils are activated to eliminate the pathogens that enter the lamina propria, and interferon-gamma (IFN-γ) further initiates the production of macrophages and dendritic cells (DC) for microbial attack. This increase in the cytokines, macrophages, and neutrophils can cause chronic inflammation, and, at this point, T_reg_ cells are very much required for the resolution purpose. The balance between T_reg_ and T_H_ cells is distorted, especially with a reduced amount of IL-10 for balancing the host–microbe ratio. Image developed using Biorender.com.

The application of high-throughput techniques like metabolomics and proteomics in combination with germ-free mice (GFM) models has emphasized the importance of microorganisms that are useful in protecting against pathogens and how an imbalance in the microbiota can induce infections [43]. Non-pathogenic commensal microbiota play a crucial role in maintaining a normal GIT physiology by ensuring effective mucosal motility, growth, immunity and nutrient digestion, absorption, and fortification of mucosal barrier [44]. Environmental and lifestyle changes, including dietary patterns, can affect the favoritism of the type of microorganism to flourish in the gut. The Western diet, with its heavy consumption of fat and sugar and its reduced dietary fiber foods often leads to the imbalance of mutualism between microorganisms in the gut. The obstruction of this relationship serves as a brilliant benefactor to the pathogenic facultative species of bacteria, like *Clostridium* spp. and *Helicobacter* spp., that overtakes the anaerobic non-pathogenic *Faecalibacterium prausnitzii* and causing dysbiosis. Rather than a pathogenic invasion, evidence suggests that microbial dysbiosis is an important factor that leads to the development of IBD [45] (Figure 3). This was found to be more pronounced in the mucosal samples than the fecal samples of CD patients collected during initial diagnosis [46]. *Faecalibacterium prausnitzii*, a beneficial microbial species that secretes anti-inflammatory metabolites, was found to be decreasing in patients with a recurrence of ileal CD [47]. *Faecalibacterium prausnitzii* can initiate the production of IL-10 and prevent the activation of inflammatory cytokines [47]. During inflammation, developed oxygen radicals can convert thiosulphate into tetrathionate to be used by *Salmonella typhimurium* as a terminal electron acceptor to obtain energy from ethanolamine [48]. The production of microcin’s by the *E. coli* strain Nissle 1917 can reduce intestinal colonization by *Salmonella enterica serovar Typhimurium* [49]. This unfamiliar shift of changing the bacterial taxa, where the promotion of the expansion of dangerous species including the Proteobacteria population and *E. coli* which can cause the exacerbation of intestinal inflammation, along with the depletion of necessary bacteria that can minimize chronic inflammation reciprocates and thereby increases the risk for colon cancer [50].

### 4.1. Mucosal Barriers and Intestinal Epithelial Cells (IECs)

The foremost type of host defense against pathogens starts with the mucus lining that represents the physical barrier covering the intestinal tract epithelium [51]. The gut contains immensely glycosylated proteins including mucin 2 (MUC2) secreted by goblet cells, protecting the proteins from degradation by the host and bacterial proteases [52]. The deficiency of MUC2 in mice leads to the destruction of the mucus layer, thus promoting increased microbiota entry to the intestinal epithelial surface and contributing to spontaneous colitis [53]. The mucosal epithelium derives its barrier capacity by utilizing an extensive network of tight junctions separating paracellular movements, which also involves dividing proteins into specific domains. The induction of mucus synthesis highly depends on the activation of Toll-like receptors (TLRs) to recognize commensal-derived products through an adaptor protein—myeloid differentiation primary response 88 gene (MYD88). The deficiency of MYD88 can lead to the lesser secretion of MUC2 [54]. TLR can initiate the activation of epithelial cells, macrophages and dendritic cells (DCs) upon pathogenic invasion, as well as initiate an inflammatory response characterized by the release of proinflammatory cytokines [55]. Interestingly, the gut epithelial cells of GFM showed reduced levels of TLR compared to normally colonized mice [56]. The inner mucus layer that is anchored to the intestinal epithelium is deprived of microbial interaction due to the presence of physical barriers, along with active antimicrobial peptides and immunoglobins. The outer layer is formed from the inner layer due to proteolytic processing of polymerized MUC2 by the bacteria that have indefinite contact with the microbiota [57]. Many microbiotas are capable of entering the epithelia in MUC2-deficient mice with an inner mucus layer [58].

The second physical barrier disconnecting the microbial ecosystem from the widely sterile tissues underneath are the intestinal epithelial cells (IECs) [51]. It produces antimicrobial peptides from the Paneth cells sensing the enteric microbial penetration of host tissues [59]. Enteric pathogens like *Salmonella enterica* are capable of evading the intestinal mucous layer by degrading the antimicrobial proteins produced by IEC and mucin by expressing genes participating in lipopolysaccharide modifications, sequestration, efflux, and degradation [60]. The IECs in the small intestine further promote segregation by secreting antimicrobial lectins, such as regenerating islet-derived protein 3γ (REG3γ), REG3β, and alpha defensin related gene that codes of cysteine rich sequence 4C (DEFCR-RS-10) that can accumulate in the mucus layer [61]. The induction of REG3γ and REG3β depends on TLR signaling, whereas DEFCR-RS-10 expression is induced by the NOD receptors [59]. Susceptibility to infection is prominent in NOD2-deficient mice, along with intestinal inflammation [62]. In addition to limiting the entry of pathogenic bacteria across the mucosal barrier, IECs can regulate the innate and adaptive immune response to promote intestinal homeostasis [2].

### 4.2. Innate Immune Cells and Myeloid Cells

Beneath the epithelium is the presence of several specialized innate immune cells like innate lymphoid cells (ILCs), DCs, macrophages, and neutrophils that complement the physical barrier of the intestinal epithelium to fight against intestinal pathogens [63]. Even though ILCs lack antigen-specific receptors, their cytokine production and transcriptional factors can mirror the three major T_H_ cells subset (T_H_1, T_H_2, and T_H_17). ILCs are categorized into three groups: ILC1s, that depend on the T_H1_- specific T box transcriptional factor T-bet or the eomesodermin gene and produce interferon (IFN)-γ; ILC2s, that rely on transcriptional factor GATA-3 and primarily produce IL-5 and IL-13; and ILC3s, which are dependent on retinoic acid receptor-related orphan receptor-γt (RORγt) and produce IL-17 and/or IL-22 [63]. Studies have shown that ILC1s can develop independently [64] of microbiota, but ILC2s and ILC3s are partially influenced by microbiota [65]. ILC1s and IFN-γ are critical in the host’s resistance to *Clostridium difficile* [66], whereas IL-13 secreted by ILC2s is critical against *Nippostrongylus brasiliensis* infection by promoting the production of mucus and inducing smooth muscle contraction [67]. ILC3s rely on the aryl hydrocarbon receptor (AhR), which is a metabolite derived from tryptophan for the production of IL-22. Certain microbes like *Lactobacillus reuteri* can metabolize tryptophan and produce ligands for AhR; they have also promoted the diminishment of the burden of *Candida albicans* intolerance in the GIT [68]. IL-22 acts on epithelial cells to mediate their barricade to maintain antimicrobial defense mechanisms in the host by reducing *Alcaligenes* spp., segmented filamentous bacteria (SFB) in the gut, and declining T_H_17 cell-mediated colitis [69]. IL-22 benefits host–microbe mutualism by promoting epithelial glycan fucosylation to promote mutualistic bacteria feeding on fructose. Any disruption in IL-22 signaling and fucosylation leads to dysbiosis, thus increasing susceptibility to enteric infections and colitis [70]. DCs are specialized antigen-presenting cells and respond to TLRs, exogenous stimuli as microbial motifs, activate T cells, and are capable of promoting immunoglobulin switching to immunoglobulin (IgA) [71].

Intestinal microbiota also promote the development and functionality of myeloid cells, which have been found to be depleted in GFM and can thus lead to pathogen invasion [72]. During intestinal damage, commensal bacteria that are diffused at the location can stimulate the synthesis of IL-1β, mainly by neutrophils to induce chemokine CXCL1 production that attracts more neutrophils to the infection site [73]. Neutrophils also synthesize anti-inflammatory mediators such as lipoxin A_4_ (LX4) as a part of resolving the inflammation [74]. Macrophages exert phagocytic and bactericidal activity but reduce the induction of pro-inflammatory cytokines to maintain intestinal homeostasis. Ly6C^high^ monocytes secrete TNF-α and IL-1β at the initial stages of inflammation, which are mediated by pathogenic microbial invasion due to dysbiosis, whereas Ly6C^low^ monocytes secreting IL-10 and TGF-β are released as a resolution phase of inflammation for control and damage repair [75].

### 4.3. T Cells and B Cells

The adaptive immune system comprises T cells and B cells that defend the GIT from intestinal pathogens [76]. Intestinal bacteria play an important task in the differentiation of T cells into various subcategories including T_H_ cells, T_H_1 cells, T_H_2 cells, T_H_17 cells, and T_reg_ cells. Upon stimulation, naïve cluster of differentiation CD4^+^ cells differentiate into T_H_ cells, thus contributing a crucial part in the initiation of an immune response [77]. T_H_1 differentiation is characterized by the release of IFN-γ, and T_H_2 initiates the production of cytokines like IL-4, IL-5, and IL-13 [78]. CD patients have increased T_H_1 cells and mucosal T_H_1/T_H_17 cells, whereas UC patients have T_H_2-cell-mediated inflammation [79]. T_H_17 cells synthesize high pro-inflammatory IL-17 and IL-22 cytokines that impart a key function in protecting against pathogens. Tumor necrosis factor ligand superfamily member 15 (TNFSF15) and IL23R are the two genes susceptible to Crohn’s disease that regulate ILC3s and T_H_17 cells and that are responsible for the induction of IL-17 and IL-22 in controlling symbiotic microorganisms [80]. NOD2 in DCs is important in inducing an early protective T_H_17 cell response in the intestinal microbiota when encountered with an enteric bacterial infection [81]. NOD2 also controls IL-23 production from DCs that, in turn, control the differentiation of T_H_17 cells [82].

T_reg_ cells synthesize IL-10, TGF-β, and IL-35, which are immunosuppressive cytokines and play fundamental tasks in maintaining tissue homeostasis between the host and the microbiota to prevent inappropriate T cell responses to microbial antigens [83].

During homeostatic conditions, the residing macrophages are inactive and do not induce an inflammatory response when encountering microbes. These inflammatory responses are regulated through IL-10 stimulation [84]. The colonization of *E. coli*, *Enterococcus faecalis*, or *Helicobacter hepaticus* can elicit colitis in IL10^−/−^ GFM [85]. The deficiency of IL-10 can also lead to the differentiation of *Helicobacter* spp.-specific CD4^+^ T cells into pathogenic T_H_ 17 and T_H_ 1 cells [86]. Symbiotic microbiota also aid in the migration of T_reg_ cells to the inflammatory damage site while inducing the expression of class A orphan G-protein-coupled receptor (GPR)15; a receptor that is necessary for the migration of T_reg_ cells [87]. Colonic T_reg_ cells can identify antigens expressed by *Clostridium* and *Parabacteroids* sp. distinctly from the T_reg_ cells of peripheral lymph nodes and the spleen [88]. Though intestinal T_reg_ cell activity can be modulated by bacterial species, the specificity of intestinal T_reg_ cells exposed to a particular microbial antigen in a complex microbiota is still unclear [89].

Plasma cells residing in gut-associated lymphoid tissues release IgA, which can promote the separation of the epithelial surface by coating bacteria and binding to microbial lipopolysaccharides, DNA, and flagellar antigens [90]. The production of IgA can be independent of or dependent on microbes through the TLRs engaged in sensing or those that are sometimes produced due to ER stress in IECs when resisting intestinal inflammation [91]. In addition to IgA production, gut microbiota like *E. coli* are also able to influence the production of IgG antibodies [92]. Patients with IBD tend to release huge quantities of IgG antibodies against symbiotic bacteria, thus increasing T cell numbers, and antibody responses support the evidence of microbiota in disease pathogenesis [91].

## 5. Nutrition

In IBD patients, protein–energy malnutrition and nutrient deficiency are prevalently linked along with other serious complications like metabolic dysfunction, secondary osteoporosis, and osteomalacia [93]. The initiation and progression of IBD can be influenced by not only the composition but also the metabolic activity of gastrointestinal microbiota that, in turn, is regulated by the host’s dietary intake including carbohydrates, protein, fat, and acute dietary changes [94]. Among dietary constituents, carbohydrates play a key role in preventing the occurrence of these diseases [95]. Carbohydrates are divided into two groups: (1) the simple sugars that are easily digested by the enzymes present in the GIT and (2) complex carbohydrates including the dietary fibers that are neither digested nor absorbed and require bacterial fermentation in the colon [96]. Fiber sources such as oat bran, protein, and guar are highly fermented, whereas cellulose, psyllium, and wheat bran are poorly fermented [97]. Notably, switching diets between fiber-rich and fiber devoid meals in 24 h was found to demonstrate a shift in bacterial diversity and resulted in fecal fermentative end-products that were microbial-derived [98]. The diets consumed by inhabitants of industrialized nations and rural nations exhibit significant differences in microbial composition [99]. Western diets include a high content of animal protein, fat, and sugar but a low fiber content, whereas the diet in rural African countries has an increased intake of fibrous food [100]. The high protein intake of meat or fish was found to be an important risk factor for IBD [101]. Dietary fibers have a significant role in improving health, specifically in mineral absorption, lipid metabolism, laxation, potential anticancer properties, and anti-inflammatory effects [102].

The rich intake of whole grains, legumes, fruits, and vegetables provides an abundant source of plant polysaccharides with a different type of glycosidic linkage in each of them. The higher the complex carbohydrates get, the more they require stronger glycosidase enzymes, which meet with the bacteria present in the colon, to metabolize [103]. The microbial conversion of complex carbohydrates/dietary fibers into simple monosaccharides involves fermentation that leads to the production of fermentative end-products called short-chain fatty acids (SCFAs), e.g., acetate, propionate, butyrate, and gases (H_2_ and CO_2_) [104]. Acetate is the most abundant SCFA (60–75%) produced by a variety of bacteria inhabiting the colon that can cross the blood–brain barrier [105]. Propionates found in the peripheral circulation [106] are produced by *Bacteroides* spp. that employs the succinate route, and bacteria belonging to the clostridial cluster IX group utilize the acrylate route from lactate. Butyrate inhibits the proliferation of colon carcinoma cells by suppressing cyclooxygenase (COX)2 expression in human colon adeno carcinoma cell line HT29 and cancer coli (CACO)-2 cancer cell lines and inducing apoptosis. Propionates protect against carcinogenesis because they can reduce the proliferation of colon cancer and differentiation via the stimulation of apoptosis and the hyperacetylation of histone proteins [107]. Propionates are also capable of inhibiting the TNFα-induced activation of NF-κB that reduces vascular cell adhesion molecule-1 (VCAM-1) expression, reduces intracellular adhesion molecule-1 (ICAM-1) expression, and increases peroxisome proliferator-activated receptor alpha (PPARα) in human umbilical vein endothelial cells (HUVEC)tissues [108]. The production of SCFAs by *Clostridia* species can provide TGF-β to help the expansion and differentiation of T_reg_ cells by enhancing IECs [109]. Thus, SCFAs produced by intestinal microbiota are significant for the differentiation of T_reg_ cells and B cells for the regulation of adaptive immune response in the GIT [110].

## 6. Conventional Therapies for IBD

**Figure 4:**
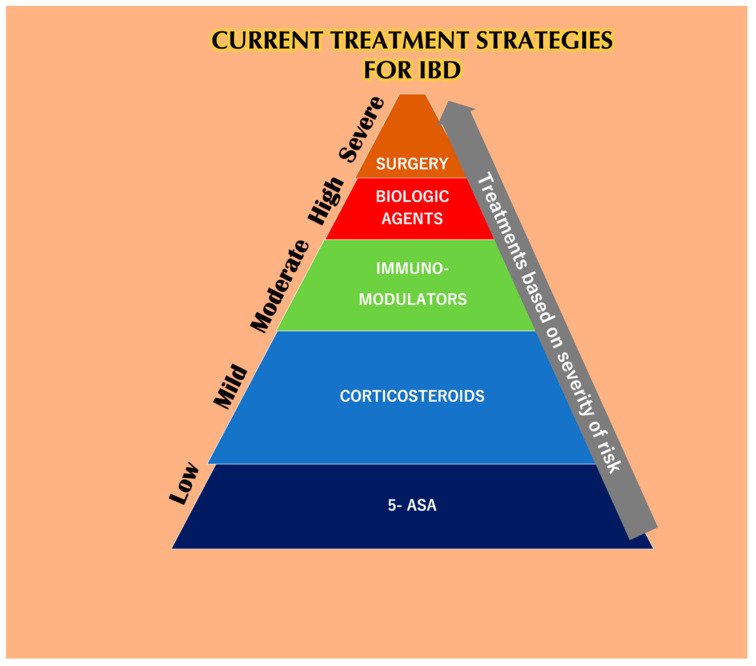
The current treatment strategies for IBD depends solely upon the severity of the disease. At lower stages of IBD, 5-aminosalicylic acid (5-ASA) helps reduce the symptoms, but not for a prolonged period because it has a reduced bioavailability. Corticosteroids are also prescribed to IBD patients in the initial stages but are burdened with systemic toxicities. Immuno-modulators are used during moderate cases of IBD but have been reported to be linked with various side effects and also lead to the risk of developing lymphoma. Biological agents like anti-TNF-α agents are used when patients are least responsive to the previous methodologies. Unfortunately, these agents are also responsible for various side effects when taken alone or as a combination with other mentioned therapies. Finally, IBD patients associated with dysplasia or cancer who undergo surgery also have a chance of relapsing [123].

The application of common therapeutics depends on the severity of the disease and the highly affected areas (Figure 4). Conventional treatment for IBD includes aminosalicylates (ASAs), systemic corticosteroids, topical corticosteroids, antibiotics, immunomodulators, and biologic therapies. ASAs for a very long time have been modified by linking 5-ASA (therapeutic moiety) through an azo bond with sulfa pyridine (carrier) to form sulfasalazine to treat IBD. ASAs are broken down in the ileocolic tract by colonic bacteria [111], boosting immunity against pathogenic bacterial antigens and also reducing inflammation by inhibiting NF-κB, IL-1, and IL-2 [112]. Unfortunately, 5-ASA and its various new preparations [4] such as olsalazine, mesalazine, and balsalazide have a poor bioavailability with various hematological side effects in addition to dose-dependent diarrhea, nausea, vomiting, abdominal pain, and fatigue [113]. Traditionally, patients with extensive mild-to-moderate active UC are given oral corticosteroids as the first line of therapy in case they fail to respond to topical mesalazine [114]. Glucocorticosteroids have also been used for the treatment of IBD, and 80% of patients have shown positive treatment responses [115]. However, the toxicity of the drugs has been linked to infections related to *Candida* spp. with an increase in blood glucose, thus compromising the glucometabolic balance in non-diabetic individuals [116]. The long term usage of corticosteroids can lead to diabetes, Cushing’s syndrome [117], and osteoporosis [118]. Budesonide and beclomethasone dipropionate (BDP) are used as topical glucocorticoids that are absorbed through the mucosa into the bloodstream and inactivated by the liver [119]. Budesonide is the first line of treatment for mildly active CD [120]. However, is not as effective as a standard glucocorticoid capable of stimulating remission in CD and also do not prevent CD relapse [118]. Budesonide was found to be less effective than systemic steroid course for inducing remission in active CD. Budesonide was also found to not be effective in the prevention of relapsing after medically or surgically-induced remission [121]. The adverse effects of budesonide are acne, weight gain, mood swings, moon face, and hair loss [122].

Antibiotics like ciprofloxacin, metronidazole, and rifaximin used for the treatment for IBD and controlling bacterial overgrowth are considered safe and well-tolerated [124]. Nonetheless, the continued treatment with ciprofloxacin can induce insomnia, acute psychosis, convulsions, dizziness, and mild-to-severe phototoxicity [125]. The adverse effects of metronidazole include anorexia, nausea, dizziness, encephalopathy, diarrhea, seizure, cerebellar ataxia, and peripheral neuropathy [126]. Common symptoms of rifaximin are nausea, abdominal pain, flatulence, vomiting, and urticarial skin reactions when consumed in high doses [127]. Thiopurines generally focus on maintenance therapy and are linked with severe side effects in more than 30% of IBD patients, along with drug discontinuation in more than 20–40% of IBD patients [128]. Thiopurines are mostly recommended for patients with steroid-dependent UC. Patients who have experienced early or frequent relapses while taking mesalazine or are intolerant depend on thiopurines. Studies have demonstrated that thiopurines can be given for at least five years in UC [114]; the most common side effects are pancreatitis, vomiting, nausea, cutaneous eruption, hepatitis [129], and a higher risk for lymphoma [130]. Methotrexate, on the other hand, is only effective in the activation and continuance of remission in CD but not in the case of UC [131], and it can induce hepatic toxicity, bone marrow suppression, gastrointestinal intolerance, and hypersensitive pneumonitis [132]. Thiopurines and methotrexate do not induce remission in severe CD, but they are considered for long term maintenance. Methotrexate is also embryotoxic and contraindicated during pregnancy because it can cause anencephaly, hydrocephaly, and meningomyelocele, thus limiting its use in young women [133]. When applied intravenously, cyclosporine A has been found to be effective in patients with severe, steroid-refractory UC [134]. However, cyclosporine A causes dose-dependent adverse effects like renal toxicity, lymphoma, hypertension, microbial infections (staphylococcus sepsis), seizures, and anaphylaxis [134].

Biological therapies including monoclonal antibodies against TNF-α and α_4_ integrins have been developed in the last 15 years to facilitate the treatment of IBD [20]. Infliximab was one of the first biologicals to be approved by the food and drug administration (FDA) for the therapy of severe, active, and fistulizing CD [135]. Infliximab can bind with mouse mTNF-α and thus induce the lysis of mTNF-α-expressing cells through antibody-dependent cellular cytotoxicity in vitro. Infliximab can also induce apoptosis in the monocytes and lamina propria lymphocytes via caspase-8, -9, and -3 in patients with CD by specifically binding to mTNF-α [136]. Studies have established that single-dose infliximab is safe and effective in the management of acute CD. Patients receiving maintenance infliximab that was used as a replacement for single-dose infliximab seemed to reduce the use of corticosteroids. Patients receiving concurrent immunosuppressive therapy developed a low incidence of antibodies against infliximab. However, the development of malignancy is linked with infliximab therapy [137]. Infusion reactions to infliximab offer multiple signs and symptoms within two hours of administration, and some of these reactions can be life-threatening. Additionally, the combination therapy of infliximab with azathioprine and methotrexate does not seem to confer an advantage over infliximab monotherapy [138]. Subsequently, the further development of anti-TNF-α antibodies like adalimumab and golimumab concerning the treatment for IBD have come into action [139] A recent study conducted on pediatric patients with CD showed that the weekly dosing of adalimumab was clinically beneficial in children who experienced nonresponse or flare on every other week dosing. Out of the 83 patients who escalated to blinded weekly dosing, 24.1% achieved remission and 51.8% achieved response. The highest rate of remission occurred those who escalated to 40 mg weekly. Abdominal and anal abscesses, as well as device-related sepsis, were observed as serious infections in patients whose doses were escalated [140]. Adalimumab and golimumab cause mild-to-moderate injection site reactions [141]. Swapping anti-TNF-α for another drug is frequently practiced when a patient becomes unreactive to one agent due to intolerance and secondary or primary failure [142]. Bone marrow toxicity (neutropenia, thrombocytopenia, and anemia) can also occur during anti-TNF-α treatment [143]. When combined with thiopurines, anti TNF-α agents were reported to cause a risk of lymphoma in patients, particularly those with CD [144]. An increased risk of opportunistic infection risk has also been associated with anti-TNF-α agents in CD patients [145]. Vedolizumab is a new immunomodulator that is effective in moderate-to-severe IBD, but it has been associated with infections, infusion-related reactions, and malignancies [146].

### 6.1. Probiotics

The complex interactions of diet, normal intestinal microbiota, and health have encouraged the introduction of probiotics that exert beneficial effects on the host [44,147]. Many microbiotas have been examined for relieving intestinal dysbiosis and bacteria like *Lactobacillus*, *Bifidobacterium*, and *Streptococcus* have been selected in the formulation of probiotics because they have shown a clinical effect on gastrointestinal inflammation and the ability to maintain normal human intestinal microbiota [148]. Probiotics have been used to understand the efficacy of living microorganisms in alleviating the symptoms of IBD [149]. VSL#3, a probiotic mixed with four strains of *Lactobacilli* (*Lactobacilli case*, *Lactobacilli acidophilus*, *Lactobacilli delbrueckii sub* spp., and *Bulgaris*), three strains of *Bifidobacterium (Bifidobacterium longum*, *Bifidobacterium breve*, and *Bifidobacterium infantis*), and a *Streptococcus* strain *(Streptococcusalivarius* sub spp. *thermophilus*) have been shown to be efficient in yielding remission in mild-to-moderately active UC [150]. One or more genetically modified strains have also been found to be more beneficial. For example, in a phase I clinical trial of treating Crohn’s disease using *Lactococcus lactis* strain, the thymidylate synthase gene exchanged using synthetic sequence encoding mature human IL-10 showed a decrease in disease activity and avoided systemic side effects [151]. Additionally, clinical trials of probiotics for the treatment of IBD are very infrequent. In an experiment conducted on 20 human volunteers (13 men and 7 women) who had a history of IBD, colon cancer, a recent antibiotic or anti-coagulant therapy was supplemented with dietary fibers and probiotics separately and in combination; the authors found no significant differences in the fecal SCFA concentrations, and no significant effect was found on epithelial proliferation [152]. *E. coli* Nissle 1917 and *Saccharomyces boulardi* probiotics did not show any significant effect in the remission of IBD and had no advantage compared with placebos [153]. Therefore, more intense understanding is required while selecting a probiotic strain when there is a disruption in the intestinal environment by disease, diet, and antibiotics that can, in turn, affect the health of the host.

### 6.2. Fecal Microbiota Transplantation

Fecal microbiota transplantation (FMT) involves the exchange of intestinal microbiota from healthy donors to re-establish intestinal microbiota in diseased individuals. FMT has been clinically adapted to recurrent *Clostridium difficile*, which leads to loss of microbiota diversity and the expansion of facultative anaerobic bacteria [154]. For the treatment of *Clostridium difficile*, FMT showed high rates of cure, regardless of the donor, recipient, and delivery method. However, some patients develop repeated infections and permanent changes in their gut microbiota upon FMT treatment for *Clostridium difficile* [155]. Clinical studies focusing on the efficacy and safety of FMT in IBD have multiplied over the years, but various factors like the selection of microbiota, immune response, and environmental factors are to be considered important factors in the pathogenesis of IBD [156]. The remission rate of FMT for patients with UC has been reported as 33%, but the long-term durability and safety are still a concern. However, there a previous study found no significant improvement in patients with CD following FMT [157]. Therefore, well-designed studies are required before randomized clinical trials in IBD [158].

## 7. The General Considerations of the Physiology of GIT during IBD for Oral Drug Delivery

The inconsistent efficacy of conventional colon-targeted deliveries is mainly due to the varied physiological factors of the GIT. The pH gradient, variable transit times, various digestive enzymes, and diversified microbial colonies are noteworthy challenges to be considered during drug delivery systems, especially through oral routes. The high acidic pH (1–2) in the stomach is required for the digestion of food, a mildly alkaline pH (6.6–7.5) is necessary for the neutralization and absorption of the food, and, finally, a very neutral pH (7–8) in the colon is especially necessary for colonic symbiotic bacteria to digest the non-starch compounds [159]. However, colonic pH gets altered in the case of IBD, with the pH going down from 5.5 to 2.3 in active UC patients [160] and to 5.3 in CD patients [161]. The change in the pH can also affect enzymatic degradation, transit time, and colonic bacterial load. Enzymatic degradation starts with the salivary amylase in the mouth, followed by pepsin and gastric lipase enzymes in the stomach, and finally trypsin enzyme in the intestines. The aerobic and anaerobic microorganisms in the colon can degrade di-, tri-, and polysaccharides due to their hydrolytic and reductive enzymes [162]. IBD results in altered physiological factors like dysmotility, increased luminal fluid (diarrhea), prolonged water secretion in the bowel, and declined reabsorption, all of which disrupt the enzymes controlling the intestinal transit that allow for nutrient absorption [163]. The decreased colonic transit time causes the poor digestion of non-starch carbohydrates, thus leading to a loss of potential symbiotic bacteria [164] and further increasing dysbiosis where pathogenic bacteria can induce inflammation [165]. Active inflammation can also alter the physiology of mucosa, and several endogenous preventive pathways are initiated in an attempt to repair the epithelial tissues. Hypoxia-inducible factor (HIF) is an oxygen-sensing transcription factor that enhances intestinal barrier function and compensates for the diminishing of mucus gel integrity with excess fluid secretion [166]. In addition, HIF transcriptionally regulates multi-drug resistance gene 1 (MDR1) which encodes P-glycoprotein (P-gp) an ATP-dependent drug efflux pump at the apical surface of cells. P-gp effluxes hydrophobic substrates like anticancer agents, steroid hormones, calcium channel blockers, and immunosuppressants that enter the member lipid bilayer from the lumen back into the extracellular medium before they reach the cytoplasm [167]. For example, glucocorticoids are substrates for P-gp and can stimulate the expression of MDR1, thus inducing steroid resistance in IBD [168]. However, nanoparticles are capable of overcoming multi-drug resistance via P-gp inhibition and ATP deletion by targeting both drug and biological mechanisms [169].

## 8. Nano Drug Delivery Systems as an Alternative

The application of nanotechnology in biomedical research arena has advanced by leaps and bounds over the past few decades, especially for cancer therapies and regenerative medicine. The unprecedented success in providing a safer alternative treatment option to current conventional cancer therapies is commendable. The extrapolation of the biomedical applications of nanotechnology or nano-drug delivery systems for the treatment of IBD is still in its infancy, though accumulating evidence has made it look promising. Since the current course of treatment for IBD-related disorders mostly comprises anti-inflammatory agents, corticosteroids, immunosuppressants, and biologic agents, these agents mainly contribute to maintaining remission from inflammatory actions, thus additionally complicating the patient profile by contributing adverse side effects. Henceforth, the discovery of alternative methods of the local delivery of these conventional agents to the inflamed tissue provides a rationale to incorporate nano-drug delivery systems in the treatment of IBD. Moreover, assistance from the use of natural compounds and conventional therapies delivered via nano-drug delivery systems could help to maintain the remission and relapse of the disease, thus improving therapeutic efficacies and avoiding systemic side-effects.

Nano-drug delivery passively or actively targets the site of inflammation and has been proven to be more beneficial than conventional therapies. Due to nano-drug delivery’s structured morphology, effective targeting, increased bioavailability, and requirement of a low concentration of the drug in unhealthy tissue, minimized, systemic adverse effects are highly anticipated [170]. The reduced size of the nanoparticles (1–1000 nm) facilitates the improved and careful transport of active molecules to the inflamed tissue via the epithelial enhanced permeability and retention effect (EPR) and promotes the selective uptake of nanoparticles by the immune cells at the target site [170]. The paracellular transport of the carrier with the drug is made permeable when the intestinal epithelial barrier is compromised [171]. The transcellular transport (transcytosis) of the nanoparticles begins with endocytosis at the cell apical membrane, followed by the release of the nanoparticles at the basolateral pole, where they come in contact with the immune cells present at the submucosal layer. The physicochemical properties of the nanoparticles (NPs), the physiology of the GIT, and the animal model are used to determine the NPs’ proper intake [172].

### 8.1. Surface Charge-Dependent Drug Delivery Systems

The surface charge of nanoparticles can be modified to influence the electrostatic interactions with the components of the GIT. Due to the presence of sulfates and sialic acid residues, colonic mucins tend to carry a negative charge. An excessively increased mucus production can be observed in CD, and this provokes a thick mucus layer in the affected area [173]. Positively charged nanoparticles can easily adhere to the negative mucosal surface within the inflamed tissues due to electrostatic interaction and thus promote cellular uptake and drug release through the better contact with the mucosal surface [174]. It was found that cationic polymethacrylate (Eudragit RL) nanoparticles (120 nm diameter) loaded with clodronate enables a complete drug release that (compared to the free clodronate) significantly decreases the myeloperoxidase activity (MPO) in the 2,4,6-trinitrobenzene sulfonic acid (TNBS) and oxazolone (OXA)-induced colitis through ionic interactions with the dissolution medium or mucin [175]. However, cationic nanoparticles have been found to have adhered to the mucosal layer and become immobilized, thus leading to the premature release of the drug, probably due to the presence of a negative mucosal surface and strong electrostatic adhesion. This was seen in the case of chitosan functionalized poly(lactic-co-glycolic acid) (PLGA) nanoparticles targeted ex vivo to intestinal mucosa adhered to the mucosal surface with minimal translocation and accumulation in both healthy and inflamed mucosa [176]. In contrast to positively charged proteins like eosinophil cationic protein and transferrin, the development of anionic nanoparticles can tackle these issues in inflamed tissues [177]. They can promote electrostatic repulsion with the negatively charged mucus, thus enabling the anionic nanoparticles to interdiffuse in the mucus network without any interactions, thus alleviating concerns regarding immobilization like in the case of cationic nanoparticles [178]. The negatively charged nanoparticles can target the inflamed mucosa and gradually release the drug depending on the microenvironment of the inflamed intestine [179]. An ex vivo study conducted on neutral, positively charged, and negatively charged liposomes to target colitis induced by dinitrobenzene sulfonic acid (DNBS) found that the adherence of anionic liposomes to inflamed colonic mucosa was two-fold more than neutral or cationic liposomes. This adherence was dependent on the negative charge on the liposomes due to the presence of 12,dimyristoyl-sn-glycerol-3-(phosphor-rac-(1-glycerol)) (DSPG), whereas cationic and neutral liposomes did not significantly bind to the inflamed intestinal mucosa [180]. Though anionic NPs are found to be specific in drug delivery, additional approaches are necessary to improve bioavailability in the colon.

### 8.2. ROS-Responsive Delivery System

Slight damage to antioxidant defense systems can lead to oxidative stress and cause an abnormal rise in the release of ROS by inflammatory cells like neutrophils and macrophages. Tackling ROS-mediated oxidative stress has been a focus for pharmaceutical strategies to improve targeted drug delivery in diseased colonic sites. Biopsies of patients with UC have found increases in mucosal ROS concentrations up to 10- to 100-fold, and redox-responsive nano-delivery systems have thus become associated with the treatment of UC [181]. Wilson et al. [182] synthesized thioketal nanoparticles formulated from polymer poly (1,4-phenylacetone dimethylene thioketal) that degrade in response to ROS for target delivery of TNF-α small interfering ribonucleic acids (siRNA)complexed with cationic species such as 1,2-dioleoyl-3-trimethylammonium-propane (DOTAP). DOTAP enhances stability in transfection, mucosal transport, and internalization inside the cell, as well as endosomal escapes to intestinal inflammation sites, in mice. Thioketal NPs diminish TNF-α messenger ribonucleic acids (mRNA) levels and protected UC. In another study, to treat colitis in mice, the oral delivery of low molecular weight TEMPOL (4-hydroxy-2,2,6,6-tetramethylpiperidin-1-oxyl) using nitroxide radical-containing nanoparticles (RNPs^O^), made up of an amphiphilic block copolymer and methoxy-poly(ethylene glycol)-b-poly(4-(2,26,6-tetramethyl-piperidine-1-oxyl)oxymethylstyrene(MeO-PEG-b-PMOT), demonstrated that the stable nitroxide radicals on the hydrophobic segment of this copolymer can successfully scavenge ROS [183]. Interestingly, RNPs^O^ were further studied to examine their effect on colonic microflora during UC, and it was found that commensal bacteria like *E. coli* and *Staphylococcus* sp. led to a remarkably high dextran sodium sulfate (DSS)-induced colitis in mice, whereas the oral administration of RNPs^O^ outstandingly reduced these commensal bacteria [184]. However, several hindrances, like the faster release of the drug and the instability of these nanocarriers in the lower pH and enzyme-rich environments of the upper GI tract, limit the application of ROS-responsive systems.

### 8.3. pH-Dependent Drug Delivery System

The pH-sensitivity of nanoparticles as a pharmaceutical strategy enables them to retain and protect their cargo, but are very likely to dissolve or swell in higher pH environments like in the colon, thus allowing for drug release [185]. When a drug directly encounters variations in pH, it becomes redundant in its activity due to extreme oxidation, deamination, or hydrolysis [186]. A synthetic polymer is often coated with pH-dependent coating polymers like methacrylic acid co-polymers (Eudragit^®^) for oral delivery [187]. Liposomes coated with Eudragit^®^ S100 display appropriate pH response release characteristics when the polymer retains the liposomal release of the drug at pH levels of 1.4 and 6.3—resembling the stomach and small intestine, respectively—but release the drug similar to plain liposomes NPs at a pH of 7.8 (ileocecal junction). However, in vivo conditions, due to the additional challenges of bile salts that cause the premature degradation of liposomes, can result in the early release of the drug in the duodenum [188]. The instability of liposomes in the GIT has pushed researchers to focus on polymer-based nanocarriers for supreme colon-specific drug delivery. Tacrolimus-loaded PLGA NPs encapsulated inside Eudragit^®^ P4135F microspheres showed zero drug or NP release at a pH of 4, but at a pH of 7.4, the NPs were released undamaged [189]. The formulation was found to be only moderately effective, and the overall poor performance was associated with the fabrication of the delivery system where the outer microspheres and the inner NPs had the same solubilities in the organic solvents. Therefore, it was difficult to check on NP integrity while being coated by the microspheres, and there are also chances for the lipophilic drug to be redeposited from the NPs into the microspheres, thus reducing the effect of the drug with partial deposition at the target site [189,190,191]. Hence, the mixing of PLGA and Eudragit^®^ was found to be more effective. The formulation of pH-sensitive NPs using PLGA and Eudragit^®^ loaded with budesonide (BSD) showed a sustained release of the drug at the colonic pH, along with more therapeutic effects than BSD alone when used to treat an TNBS-induced animal model of colitis [192]. Beloqui et al. [187] prepared nanoparticles using pH-sensitive PLGA and Eudragit^®^ S100 loaded with the anti-inflammatory agent curcumin, which is preferential in accumulating at the inflamed region in in vitro and in vivo studies. The permeation of the drugs was found to be enhanced by curcumin-loaded NPs across Caco-2 monolayers and also reduced TNF-α secretion through LPS-activated macrophages (J774 cells) in comparison to the curcumin suspension. In in vivo conditions, NPs were found to significantly reduce neutrophil infiltration while retaining a colonic structure identical to the control group in a murine DSS-induced colitis model. Ribeiro et al. [193] fabricated a drug delivery system by coating pectin on chitosan/layered double hydroxide biohybrid beads loaded with 5-ASA for protection against degradation at the upper GIT. Coating with pectin was able to navigate through the gastric juices and promote the release of the drug from the bio-nano composite beads due to swelling of pectin at pH 7.4. Cyclosporine loaded in PLGA coated with Eudragit^®^ S100 nanospheres were able to generate a sustained release at a pH of 7.4, thus suggesting its capability in UC therapy [194]. Additionally, pH-sensitive Eudragit^®^ S100/ethylcellulose nanofibers loaded with budesonide showed the supreme release of the drug at a pH of 7.4, which was similar to that of spherical NPs [195]. In another in vitro analysis, 5-ASA-loaded chitosan NPs coated with Eudragit^®^ S100 revealed that the drug release was only at the pH values of the colon [196]. Chitosan and alginate coated with Eudragit^®^ S100 pH-sensitive microcrystals also illustrated pH-dependent dexamethasone release, avoiding drug release in the acidic pH conditions of the stomach and small intestine. This enabled the release of the drug in the colonic pH and alleviated inflammation in a DSS-induced mouse colitis model [197]. Though pH-dependent NPs have shown tremendous results in preclinical studies, the variability of pH in IBD patients’ colons shows that a colonic drug delivery system based only on GIT pH would not be reliable [198].

### 8.4. Biodegradable Drug Delivery Systems

When developing a drug delivery system, the chief goal is to protect the hydrophobic therapeutically active molecules prematurely subjected to degradation for enhancing sustained release at the targeted site and to avoid causing an undesired side effect. One of the ideal methods would be controlled-release systems that could maintain the drug concentration and frequency of administration [199]. Polysaccharides like pectin, chitosan, and alginate have been studied for the oral delivery of hydrophobic drugs for targeting inflammation in the colon [200]. High-water content hydrogel is a cross-linked polymer network that provides physical similarity to biological tissues and thus has an exceptional biocompatibility. Hydrogels can encapsulate hydrophilic drugs with minimal denaturation and aggregation upon exposure to organic solvents [201]. Laroui et al. [202] developed a hydrogel using chitosan and alginate that was cross-linked using Ca^2+^ and SO^2-^ to encapsulate polylactic acid (PLA) NPs with the anti-inflammatory tripeptide Lys–Pro–Val (KPV). Upon reaching the inflamed colon, the hydrogel was degraded and successfully reduced colitis symptoms, MPO activity, and histologic alterations in a DSS colitis model. In vivo studies have indicated that microparticles made of resistant starch such as high amylose cornstarch loaded with 5-ASA have a high tolerance against the acidic and enzymatic conditions of the upper GIT and can accurately release the drug in the colon [203]. A nanoparticle-in-microparticle oral delivery system (NiMOS) was also designed for colon targeting by encapsulating plasmid and siRNA in type B gelatin nanoparticles. These nanoparticles were loaded in poly(epsilon-caprolactone) (PCL) microspheres that can withstand protein/enzymatic degradation in the upper GIT. The NP release occurs at the inflamed sites of the intestine due to the action of the lipase enzyme on PCL present at the location [204]. Pectin-based microspheres/nanospheres resonate a viable oral colon-specific drug carrier since the gut bacteria like *Bacteroides thetaiotamicron* and *E. coli* are capable of degrading the pectin in the colon [205]. Crosslinked pectin microspheres loaded with indomethacin in vitro showed an increased delivery at pH 7.4 compared to non-crosslinked microspheres. Similarly, Eudragit-coated pectin microspheres were also found to be excellent in the colon-specific delivery of the drug [206]. In a recent study, mesalazine-loaded calcium pectin–silica gel beads were developed to control the release of mesalazine in the colon. These beads showed a reduced delivery of mesalazine in a simulated upper GIT condition due to decreased swelling that, in turn, improved the strength of the bead. An elated drug level was found in the simulated colonic fluid with an increased sensitivity of pectin towards the pectinase [207]. In another study, Eudragit^®^ FS30D-coated alginate microspheres filled in hydroxypropyl methylcellulose (HMPC) capsules ensured the release in the colon even though Eudragit FS30D had a solubility before reaching a pH of 7. In vitro studies in simulated colonic fluid with rat fecal content confirmed the bacterial degradability of the alginate, thus prematurely hindering the drug release in the upper GIT. In vivo studies have also shown a marked reduction in the ulcer index in rats treated with microspheres [208]. Resveratrol is a naturally therapeutic agent, but it is also a hydrophobic drug, and the necessity of a hydrophilic carrier is therefore of utmost importance. Biocompatible and non-toxic poly(2-hydroxyethyl methacrylate) and a pH-sensitive poly(*N*,*N*-dimethylaminoethyl methacrylate) loaded with resveratrol were integrated into a chitosan matrix gel. The drug was released in a sustained release pattern due to the presence of the chitosan network, thus proposing a versatile tool that can bestow therapeutic benefits in the treatment of IBD [209]. An acetic acid-induced colitis rabbit model was used to study the effect of the quercetin drug, a natural polyphenol in a chitosan-based colon targeted delivery system to selectively target the inflamed colon. The drug-loaded microparticles were more therapeutically effective than a plain drug [210].

Edible plant-derived nanoparticles have also been designed for a novel and nontoxic delivery system to target colon tissues, thereby reducing IBD-mediated inflammation [208]. Zhang et al. [211] developed grapefruit-derived nanovesicles loaded with methotrexate that are biodegradable, biocompatible, and stable across a wide range of pH conditions. The methotrexate nanovesicles were able to downregulate IL-1β and TNF-α by upregulating the release of heme oxygenase-1 (HO-1) in intestinal macrophages and had improved anti-inflammatory properties against DSS-induced IBD compared to a free drug. The oral administration of ginger-derived nanoparticles was also found to increase IEC proliferation and elevate the concentration of anti-inflammatory cytokines by reducing the concentration of proinflammatory cytokines like TNF-α, IL-1β, and IL-6 [212]

### 8.5. Active Targeting-Dependent Nano Delivery Systems

Active targeting-dependent nano-delivery systems prioritize the parenteral route for various conditions like cancer, infections, and inflammation [213]. This type of targeting has been proven to be precise in the case of spatial localization, increasing the therapeutic efficacy of the drug and reducing toxic effects on normal tissues [214]. The promising results of this research have led to the exploitation of this strategy in oral nano-drug delivery systems. CD98 is a glycoprotein that is upregulated in colonic epithelial and macrophage cells in the inflamed state of UC. The oral administration of nanocarriers encapsulating anti-CD98 siRNA was found to reduce CD98 expression in DSS-induced colitis in mice [215]. The expression of ICAM-1 in the colonic epithelium during inflammation is quite common in IBD [216]. Mane and Muro [217] examined the GIT biodistribution, cellular uptake, and degradation of ICAM-1 antibody-coated polystyrene nanoparticles in wild-type C57BL/6 mice by utilizing radiolabeling and fluorescence; of the total dose delivered, ~60% were susceptible to GIT enzymatic degradation, and most of the NPs were deposited in the stomach and duodenum, thus suggesting upper GIT targeting. Saccharides including mannose, galactose, and hyaluronic acid could be used in active targeting [214]. Xiao et al. [218] formulated a mannosylated bio-reducible cationic polymer to form NPs via the electrostatic interaction of sodium triphosphate (TPP) and TNF-α siRNA. The NPs displayed potential effects in reducing TNF-α in a DSS-induced colitis model, both in vitro and ex vivo. In a different study, the expression of mannose receptors on the macrophage membrane was exploited via the loading of ovalbumin in mannosylated PLGA nanoparticles, which were also able to tremendously accumulate in the inflamed tissues [20]. The galactose receptor has also been targeted for an oral nano-delivery system. Mitogen-Activated Protein Kinase Kinase Kinase Kinase 4 Map4k4 siRNA uptake using galactosylated trimethyl chitosan TMC cysteine nanoparticles successfully suppresses DSS-induced UC in mice by constraining the initiation of TNF-α production [219]. Hyaluronic acid-functionalized polymeric NPs containing CD98 siRNA and curcumin was able to focus on the targeted delivery of the drugs to the key cells related to UC therapy, prevent mucosal damage, and reduce DSS-induced inflammation by inhibiting the over-expression of CD98 and TNF-α [220]. Compared to a free drug, a biocompatible hyaluronic acid based-nanoparticulate carrier loaded with budesonide showed an elevated anti-inflammatory effect on proinflammatory cytokine secretion in IBD [221]. A naturally occurring protein like lecithin is highly specific for carbohydrate residues and has been extensively applied in colon-specific drug delivery as a ligand. Moulari et al. [222] developed peanut and wheat germ lectin-decorated NPs to selectively deliver the drugs to inflamed tissues. Ex vivo quantitative adhesion analyses showed that lectin decorated nanoparticles had superior binding and selectivity to inflamed tissues in comparison to NPs. Naserifar et al. [223] studied the effect of resveratrol, a natural stilbenoid with excellent anti-inflammatory properties, for IBD; resveratrol was loaded in folic acid-conjugated PLGA NPs to induce targeted delivery to enterocytes. The oral administration of the NPs to a TNBS-induced rat model successfully demonstrated the reduction of colitis in vivo.

Antibodies as ligands in nanocarriers for selective targeting and delivery can be used in their native states or as fragments for binding to receptors that are highly expressed on the diseased cell surface [224]. This binding leads to receptor-mediated internalization, which allows the nanocarriers to release the drug inside the cell [225]. The transferrin receptor protein is modestly expressed in healthy tissues but was highly expressed in the basolateral and apical membranes of enterocytes in the colonic mucosa of IBD patients, as well as the colonocytes of rats induced with colitis [226]. Ex vivo binding studies utilizing anti-transferrin receptor immune liposomes have found higher concentrations in the mucosa of rats with DNBS-induced colitis than non-conjugated immunoliposomes [226]. CD98 is a heterodimeric neutral amino acid transporter that is suitable for active targeting. Mice with active colitis have shown the overexpression of CD98 by intestinal B cells, CD4^+^T cells, and CD8^+^T cells [227]. When orally administered, CD98 siRNA/polyethyleneimine (PEI)-loaded NPs in a hydrogel were found to be non-toxic and biocompatible, and they also reduced CD98 expression and colitis in mice [215]. The epithelial growth factor -like module containing mucin like hormone receptor like 1 also known as F4/80 monoclonal antibody is also a vital macrophage-specific marker used in separating macrophages [228]. A poly (lactic acid) poly (ethylene glycol) block copolymer (PLA-PEG) grafted with the Fab’ portion of the F4/80 antibody (Ab) was used in the encapsulation of TNF-α siRNA to form TNF-α siRNA-loaded PLA-PEG NPs. The oral administration of NPs attenuated DSS-induced colitis and revealed F4/80 Fab’-functionalized NPs better than plain NPs. Additionally, flow cytometry analyses demonstrated that F4/80 Fab’-functionalized NPs enhanced the macrophage-targeting ability and endocytosis of NPs [229]. On the whole, these studies indicated that active targeting is a powerful potential strategy for improving targeted drug delivery and uptake in the case of IBD. However, improved understanding using in vivo experiments that assess different targeting ligands are necessary for an improved delivery system in animal models of colitis. Table 2 depicts the tabular representation of a few examples of NPs described in the text.

## 9. Conclusions and Perspectives

IBD is a global disease that is steadily sweeping across international borders from the Western world and expanding into newly industrialized countries in Asia, Africa, and the Middle East [230]. In this review, we discussed the impact of genetic factors and the relationship between the gut immune system and the microbiome that has potential for the development of IBD. The direct link of various genes like NOD and the co-existing relationship between intestinal microflora and the immune system in the pathophysiology of IBD remain unanswered questions. The conflict of dysbiosis in genetically susceptible individuals is often with exposure to new environmental factors that may directly or indirectly affect each patient. The diversity of the characteristics of the disease leads to failures in understanding the excellent course for treatment. Patients with IBD are also at the risk of developing colorectal cancer due to inflammation and immunosuppression. Conventional medical therapies are beneficial but often associated with severe systemic side effects and complications. The rapid evolution of “omics” technologies promises the importance of understanding the effect of interspecies variations in the symbiont ecosystem present in the intestines so that researchers can carefully differentiate good bacteria from the bad bacteria involved in the pathology of the disease [231]. The beneficial effects of probiotics tailored for each individual at the cost of their varied genetic makeup, along with diet patterns, are still debated due to conflicting research. Further studies on overcoming these barriers are still in progress.

The design of a nano-drug delivery system has swept the pharmaceutical field with advanced results in IBD therapy focusing on selective targeting, effective drug localization with reduced systemic side-effects, and toxicity. This has revolutionized the use of hydrophobic compounds with poor bioavailability to be utilized in oral delivery. However, the acceptance of the translation of nanocarrier systems into clinal trials is still limited due to various barriers, like the safety of multiple nanoparticles and the specificity of the ligands used for active targeting, that still need proper addressing. Other issues still under research are the premature release of the drug before reaching the target site, enzymatic degradation in the upper GIT, the large-scale production of nanocarriers, and inter-patient variation in human IBD. Biomimetic nanoparticles developed through nanoengineering from human immune cells like neutrophils and macrophages support the mimic methodology of the cells exacerbated at the inflamed site and support selective targeting for effective delivery. Currently, multidrug delivery and multi sophisticated nanocarrier systems are on the rise because they can respond to both pH and enzymatic degradation. Though in vitro and ex vivo stability, binding and uptake have been thoroughly researched, though a well-established colitis model is required to validate the same parameters in vivo. Animal models of IBD also play a crucial role in screening the intriguing roles of genes, microbiota, and the immune system in the course of the disease. However, these models do not fully link to the genetic defects associated with human disease and can hinder the decoding of the actual pathology of IBD. The microbiota of the gnotobiotic mice commonly used for experimental purposes are often simplified and cannot be compared to the complex microbe–host relationship seen in the human GIT. Thus, the necessity to develop models relevant to human IBD is very crucial because only then will the practicability of designing a tailored dosage form of this incredible technology become a reality.

## Figures and Tables

**Table 1:** Comparison between Crohn’s disease (CD) and ulcerative colitis (UC).

Parameters	UC	CD
Gender [26,27]	Males	Females
Onset [28]	30–40 years of age	20–30 years of age
Site	Distal colon [3]	Any part of the gastrointestinal tract (GIT) [2]
Complications	Cryptitis, fistulas, severe bleeding [3], and colon cancer	Strictures, abscesses, fistulas [2], colon cancer
Smoking	Protective role-reduced colectomy and sclerosing cholangitis [29]	Threatening—leading to gut irritation, an impaired immune response, and respiratory disease [30]
Post-Surgery	Surgically curable but higher risk for adults above 50 years due to postoperative complications like infections and abscesses [31]	Relapse even after the surgical removal of the affected portion and postoperative complications like short bowel syndrome [32]

**Table 2:** Few examples of therapeutic nanoparticles/microparticles that have experimentally (in vitro, in vivo, and ex vivo conditions) used in the treatment of IBD.

Nanoparticles	Therapeutic Agent	Characteristics
Cationic Polymethacrylate (Eudragit RL) NPs	Clodronate	Decreased MPO activity in TNBS- and OXA-induced-colitis compared to free clodronate [175]
Polymeric (poly 1,4-phenyl acetone dimethylene-thioketal) NPs complexed with DOTAP	TNF-α small interfering ribonucleic acid (siRNA)	Thioketal NPs degrade in response to ROS, and DOTAP enhances mucosal transport. Protects UC by decreasing TNF-α level [182]
Nitroxide-radical NPs comprising of block copolymer methoxy-poly(ethylene glycol)-b-poly(4-(2,26,6-tetramethyl-piperidine-1-oxyl)oxymethylstyrene (MeO-PEG-b-PMOT)	TEMPOL	Reduction in colitis with successful scavenging of ROS [183]
Poly(lactic-co-glycolic acid) (PLGA) NPs encapsulated inside Eudragit P4135F microspheres	Tacrolimus	Provided protection to the drug at acidic pH [189]
PLGA/Eudragit NPs	Budesonide (BSD)	Sustained release of BSD at colonic pH and improved therapeutic effects of BSD against TNBS-induced colitis [192]
PLGA/Eudragit S100 NPs	Curcumin	Reduced TNF-α secretion in LPS-activated macrophages. Reduced neutrophil infiltration in a DSS-colitis model [187]
Pectin coated-chitosan-hydroxide biohybrid beads	5-ASA	Protection to NPs to transport through gastric juices in upper GIT and sustained release of the drugs at 7.4 [193]
PLGA-coated Eudragit S100 nanospheres	Cyclosporine	Sustained release at 7.4 and used in UC therapy [194]
Eudragit S100/ethyl cellulose nanofibers	Budesonide (BSD)	Sustained release of drug at a pH of 7.4 [195]
Chitosan and alginate coated with Eudragit S100 microcrystals	Dexamethasone	Release of drug in colonic pH and alleviate inflammation in DSS-colitis model [197]
Chitosan and alginate hydrogel/PLA NPs	Anti-inflammatory tripeptide Lys–Pro–Val (KPV)	Reduced MPO activity and colitis symptoms [202]
Cross-linked pectin microspheres	Indomethacin	Increased colon-specific delivery at a pH of 7.4 [206]
Calcium pectin–silica gel beads	Mesalazine	Controlled release of mesalazine at the simulated upper GIT condition and increased mesalazine release in the simulated colonic fluid [207]
Grapefruit-derived nanovesicles	Methotrexate	Downregulation of IL-1β and TNF-α by upregulating release of heme-oxygenase I and induced anti-inflammatory properties against DSS-IBD [211]
Ginger-derived NPs	Ginger	Elevated anti-inflammatory cytokines and downregulated concertation’s of pro-inflammatory cytokines TNF-α, IL-1β, and IL-6 [212]
PEI NPs	Anti-cluster of differentiation (CD)98 siRNA	Reduction of CD98 expression in DSS-induced colitis [215]
Mannosylated bio-reducible cationic polymeric NPs	TNF-α siRNA	Reduction in TNF-α expression in DSS-induced colitis [218]
Galactosylated trimethyl chitosan cysteine NPs	Mitogen-Activated Protein Kinase Kinase Kinase Kinase (Map4k4) siRNA	Blocks the TNF-α production in DSS-induced UC [219]
Peanut and wheat germ-lecithin decorated NPs	Betamethasone	Specificity towards inflamed tissues having high expression of lecithin [222]
PLA-PEG (poly (lactic acid) poly (ethylene glycol) block copolymer) grafted with the Fab’ portion of F4/F80 Ab	TNF-α	Enhanced macrophage-targeting and endocytosis of NPs. Oral administration led to attention in DSS-induced colitis [229]
Folic acid-conjugated PLGA NPs	Resveratrol	Oral delivery reduced colitis in TNBS-induced rat model [223]
Chitosan microparticle	Quercetin	Oral administration reduced colitis in an acetic acid-induced rabbit model compared to free quercetin [210]

Abbreviations used: MPO: myeloperoxidase; TNBS: 2,4,6-trinitrobenzene sulfonic acid; OXA: oxazolone; DOTAP: 1,2-dioleoyl-3-trimethylammonium-propan; TNF-α: tumor necrosis factor; IL: interleukins; ROS: reactive oxygen species; UC: ulcerative colitis; TEMPOL: 4-hydroxy-2,2,6,6-tetramethylpiperidin-1-oxyl; LPS: lipopolysaccharide; DSS: dextran sulfate sodium; 5-ASA: aminosalicylic acid; GIT: gastrointestinal tract; NP: nanoparticle; PEI: polyethyleneimine.
